# Transforming Growth Factor-β1 Suppresses Hepatitis B Virus Replication by the Reduction of Hepatocyte Nuclear Factor-4α Expression

**DOI:** 10.1371/journal.pone.0030360

**Published:** 2012-01-20

**Authors:** Ming-Hsiang Hong, Yu-Chi Chou, Yi-Chieh Wu, Kuen-Nan Tsai, Cheng-po Hu, King-Song Jeng, Mong-Liang Chen, Chungming Chang

**Affiliations:** 1 Institute of Microbiology and Immunology, National Yang-Ming University, Taipei, Taiwan; 2 Institute of Molecular and Genomic Medicine, National Health Research Institutes, Miaoli, Taiwan; 3 Institute of Molecular Biology, Academia Sinica, Taipei, Taiwan; 4 Institute of Molecular Medicine, National Tsing Hua University, Hsinchu, Taiwan; 5 Department of Life Science, Tunghai University, Taichung, Taiwan; 6 Center for Molecular Medicine, China Medical University and Hospital, Taichung, Taiwan; Yonsei University, Korea

## Abstract

Several studies have demonstrated that cytokine-mediated noncytopathic suppression of hepatitis B virus (HBV) replication may provide an alternative therapeutic strategy for the treatment of chronic hepatitis B infection. In our previous study, we showed that transforming growth factor-beta1 (TGF-β1) could effectively suppress HBV replication at physiological concentrations. Here, we provide more evidence that TGF-β1 specifically diminishes HBV core promoter activity, which subsequently results in a reduction in the level of viral pregenomic RNA (pgRNA), core protein (HBc), nucleocapsid, and consequently suppresses HBV replication. The hepatocyte nuclear factor 4alpha (HNF-4α) binding element(s) within the HBV core promoter region was characterized to be responsive for the inhibitory effect of TGF-β1 on HBV regulation. Furthermore, we found that TGF-β1 treatment significantly repressed HNF-4α expression at both mRNA and protein levels. We demonstrated that RNAi-mediated depletion of HNF-4α was sufficient to reduce HBc synthesis as TGF-β1 did. Prevention of HNF-4α degradation by treating with proteasome inhibitor MG132 also prevented the inhibitory effect of TGF-β1. Finally, we confirmed that HBV replication could be rescued by ectopic expression of HNF-4α in TGF-β1-treated cells. Our data clarify the mechanism by which TGF-β1 suppresses HBV replication, primarily through modulating the expression of HNF-4α gene.

## Introduction

Chronic infection with hepatitis B virus (HBV) is a major worldwide issue in public health and is one of the best known high-risk factors for cirrhosis and hepatocellular carcinoma (HCC) [1], [2]. The molecular mechanisms of HBV replication and the regulatory elements within the HBV genome have been extensively studied [3], [4]. Following virus entry into hepatocytes, the circular partially double-stranded viral genome is converted into a covalently closed circular DNA (cccDNA) in the nucleus. This cccDNA then becomes the template for transcription of pregenomic RNA (pgRNA) and other subgenomic messenger RNAs (mRNAs). The viral pgRNA not only serves as mRNA for the synthesis of the HBV core protein (HBc) and polymerase but also assembles with the HBc and the viral polymerase to form nucleocapsids [3]. Thus, the expression level of pgRNA is considered to play a central role in controlling the level of HBV replication [5].

The transcription of pgRNA is regulated by the core promoter, which consists of the basal core promoter and the core upstream regulatory sequence (CURS, also referred as enhancer II) [6]. The CURS contains several cis-acting elements and the binding of different transcription factors on this region positively or negatively modulates the downstream core promoter activity [4], [7]. Several transcription factors, including specific protein 1 (SP1), chicken ovalbumin upstream promoter transcription factor 1 (COUP-TF1), hepatocyte nuclear factor 3 (HNF-3), HNF-4α, human testicular receptor 2 (TR2), peroxisome proliferators activated receptor alpha (PPARα), and retinoid X receptor alpha (RXRα) have been shown to interact with the CURS region of the core promoter [8], [9], [10]. Among these transcription factors, HNF-4α plays an important role in controlling the expression of viral pgRNA. The binding of HNF-4α to the regulatory element of the core promoter has been shown to generate the fundamental complex required for pgRNA synthesis [5], [11]. Moreover, HNF-4α is reported to differentially regulate transcription of pgRNA and pre-C mRNA. Overexpression of HNF-4α alone leads to at least an eight-fold increase in pgRNA synthesis but only a two-fold increase in pre-C mRNA [12]. Moreover, HNF-4α plays a transcriptional hierarchy which controls hepatic genes expression, hepatocyte differentiation, and even liver morphogenesis [13], [14], [15], [16].

Transforming growth factor-beta 1 (TGF-β1) is a pleiotropic cytokine that is implicated in multiple biological functions in the liver, including the delay of hepatocyte proliferation, the modulation of hepatocyte growth factor signaling, and the apoptosis of HCC [17]. The expression level of TGF-β1 is significantly increased after liver injury, during which hepatic stellate cells (HSCs) are considered to be the major source of secreted TGF-β1 [18]. Moreover, it has been reported that HBV replication induces hepatocytes to secret TGF-β [19]. Clinical investigation also revealed that plasma TGF-β1 is significantly elevated in patients with chronic hepatitis (CHB), cirrhosis, and HCC [20], [21], [22]. In our previous study, we have shown that TGF-β1 also functions as an antiviral cytokine which could efficiently inhibit HBV replication in HBV-producing HepG2 cells [23]. However, the precise molecular mechanism by which TGF-β1 exerts its antiviral effect is not well characterized.

Here, we provide more evidence to clarify the molecular mechanism of HBV inhibition mediated by TGF-β1. We demonstrated that TGF-β1 represses the expression of HNF-4α, which reduces the level of HBV pgRNA and consequently inhibits HBV replication. Our data suggests that HNF-4α is the key responsive regulator in TGF-β1-mediated HBV suppression. The detail mechanism by which TGF-β1 inhibits HBV replication and the role of HNF-4α in cytokine-mediated viral clearance are discussed.

## Results

### TGF-β1 represses HBV replication in the stably HBV-expressing HepG2 cells

**Figure 1:**
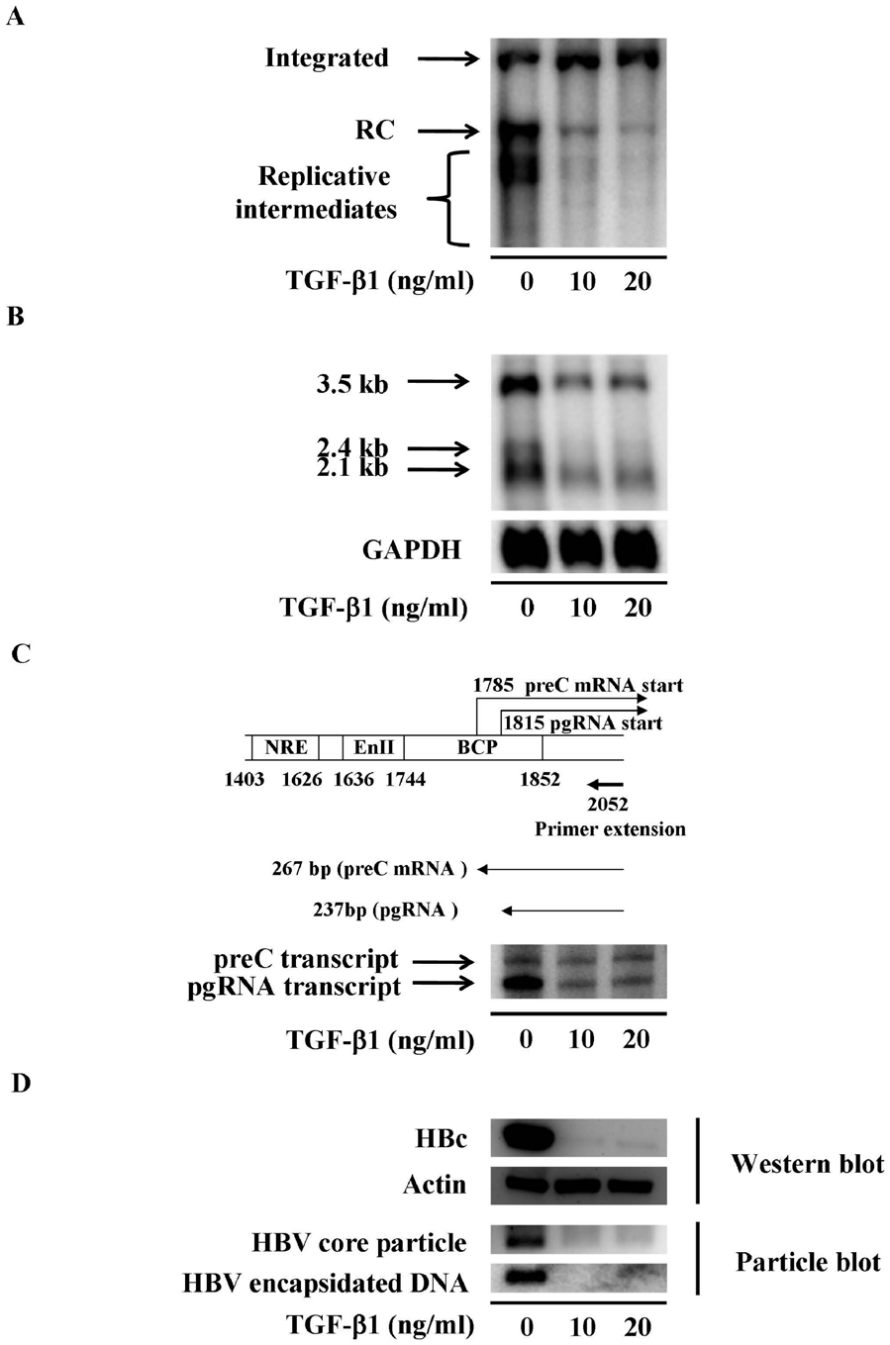
TGF-β1 represses viral replication in HBV expressing cells. 1.3ES2 cells were treated with or without TGF-β1 for 6 days. (A) Total DNA was extracted followed by HindIII digestion. The viral replicative intermediates were examined by Southern blot analysis using an HBV-specific probe. The intensity of the integrated HBV genome was used as internal control for the equal amount of sample loading. Bands corresponding to the integrated genome (integrated), the relaxed circular double-stranded DNA (RC), the replicative intermediates are indicated individually. (B) Total RNA was extracted, and the expression of viral transcripts were examined by Northern blot analysis using HBV-specific probe. Expression of GAPDH was used as an internal control. (C) The amount of pgRNA and pre-C transcripts was analyzed by primer extension analysis. The schematic illustration shows the relative position of the HBV-specific primers used for primer extension analysis. (D) The cell lysate was harvested, and the expression level of HBc was determined by Western blot analysis. HBV nucleocapsids and the embedded viral gnome were detected by using particle blot analysis. Expression of actin was used as an internal control.

To elucidate the effects of TGF-β1 on HBV replication, 1.3ES2 cells were treated with or without TGF-β1 for 6 days, and the inhibitory effects of TGF-β1 on HBV replication were examined. As mentioned in our previous study [23], the expression levels of viral relaxed circular double-stranded DNA (RC) and replicative intermediates were substantially repressed in the presence of TGF-β1 (Fig. 1A). Meanwhile, the synthesis of viral transcripts, especially the 3.5 kb transcripts of HBV, was efficiently repressed by TGF-β1 treatment (Fig. 1B). To clarify which species of HBV 3.5 kb transcripts (either pre-C mRNA or pgRNA) was reduced by TGF-β1 treatment, we performed primer extension analysis to detect the amounts of pre-C mRNA and pgRNA in the presence of TGF-β1. An HBV-specific primer was used for the primer extension assay, which was designed to generate a 237-nucleotide fragment from pgRNA and a 267-nucleotide fragment from pre-C mRNA. By densitometry analysis, our data revealed that treatment with 10 ng/ml of TGF-β1 significantly inhibited the level of pgRNA by 81% but only diminished pre-C mRNA by 16% (Fig. 1C). The differential reduction of pgRNA and pre-C mRNA by TGF-β1 treatment was consistent with our previous finding [23]. The preferential reduction of HBV pgRNA by TGF-β1 treatment suggested that the expression level of HBc should be specifically repressed. Indeed, we found by Western blot analysis that TGF-β1 treatment dramatically decreased the level of HBc (Fig. 1D). On the other hand, the expression level of HBV e antigen (HBe), which was translated from pre-C transcripts, was not profoundly influenced under TGF-β1 treatment ([23] and data not shown). Furthermore, Particle blot analysis demonstrated that the formation of nucleocapsids was significantly repressed by TGF-β1 treatment, as shown by the disappearance of both viral capsids and encapsidated nucleic acids after TGF-β1 treatment (Fig. 1D). These results indicated that TGF-β1 treatment specifically reduced the expression of viral pgRNA, which subsequently decreased the level of HBc, prevented the assembly of intracellular nucleocapsids and finally blocked HBV replication. We therefore further clarified the mechanism by which TGF-β1 exerts its inhibitory effect on HBV replication.

### The HNF-4α binding sites are the responsive elements for the repression of HBV core promoter activity by TGF-β1

**Figure 2:**
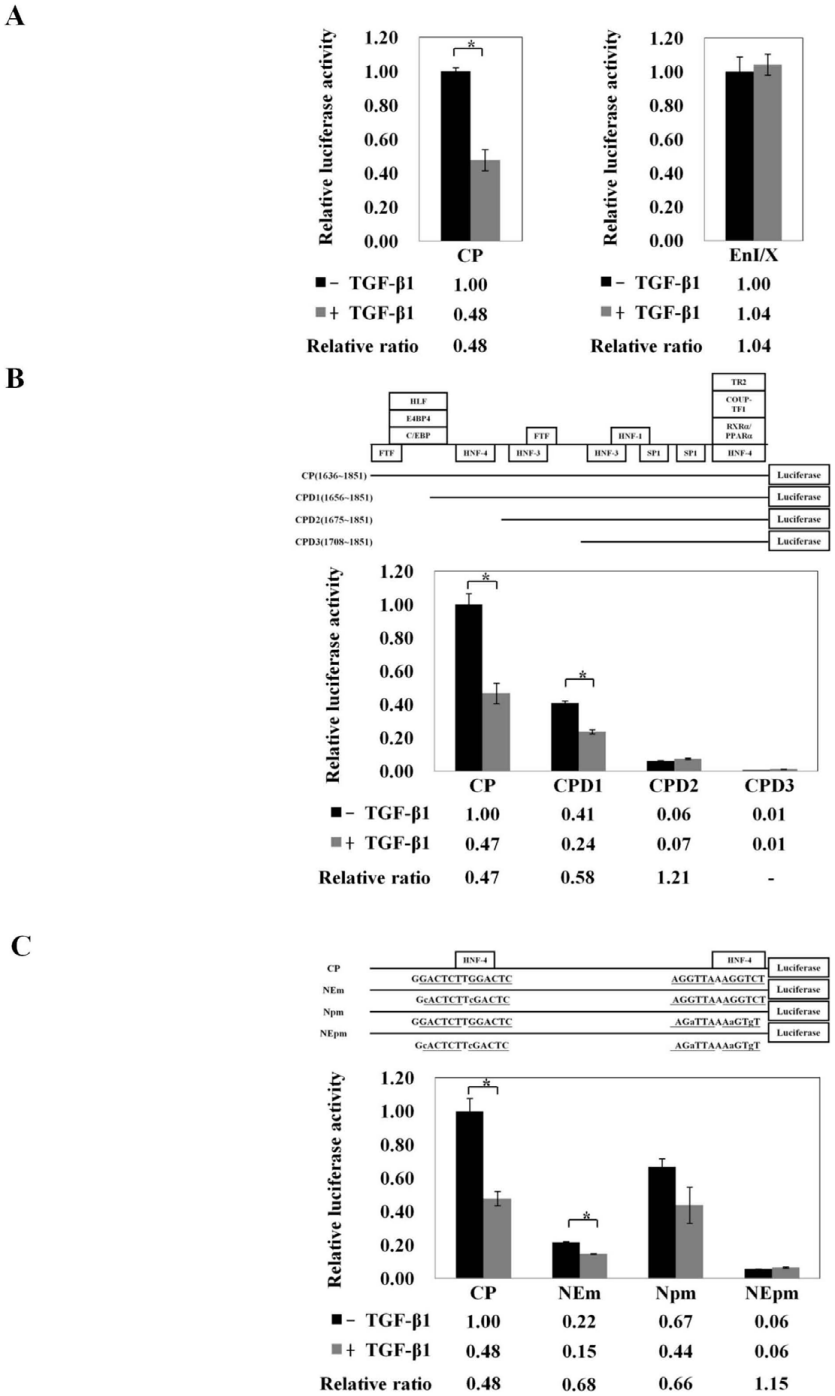
The HNF-4α binding sites within HBV core promoter are the essential elements for the inhibitory effects of TGF-β1. For the promoter activity assay, HepG2 cells were transfected with luciferase reporter plasmids driven by HBV core promoter (CP) and enhancer I/X promoter (EnI/X) (A), with luciferase reporter plasmids driven by HBV core promoter (CP) and truncated HBV core promoters (CPD1, CPD2 and CPD3) (B), or with luciferase reporters with HNF4BE(s)-mutated HBV core promoters (NEm, Npm and NEpm) (C). Two days after TGF-β1 treatment, the cell lysate was extracted for luciferase activity analysis. The luciferase activities from cells transfected with different reporter plasmids were normalized to the galactosidase activities from co-transfected pCMV-beta-galactosidase plasmids, and the relative luciferase activities were compared to that of core promoter (CP) or enhancer I/X promoter (EnI/X), respectively. The relative ratio of their promoter activities between TGF-β1-treated cells and corresponding mock control cells were shown below. The schematic illustration shows the reporter constructs with deletions or specific mutations of HNF-4α binding sites in the HBV core promoter region. The results were shown as relative ratio of the firefly luciferase activities normalized to the beta-galactosidase activities in triplicates (mean value±S.D). Statistical analyses were carried out by 2-sided paired t test, and the star symbol representing the *p* values less than 0.01 were considered as statistically significant.

Previous studies have identified several regulatory elements which are located within the enhancer I/X promoter and core promoter region contribute to the transcriptional regulation of pgRNA [4], [7], [24], [25]. To explore the responsive element involved in the TGF-β1-mediated reduction of HBV pgRNA, HepG2 cells were transfected with luciferase reporters, which contains HBV enhancer I/X promoter and core promoter, and the promoter activities were measured in the presence or absence of TGF-β1. We found that TGF-β1 specifically and substantially inhibited HBV core promoter by 52% (Fig. 2A, left panel, CP). However, TGF-β1 only slightly modulated enhancer I/X promoter. (Fig. 2A, right panel, EnI/X). To further investigate the responsive element involved in the TGF-β1-mediated reduction of HBV pgRNA, HepG2 cells were transfected with a series of luciferase reporters, which contains intact HBV core promoter (Fig. 2B, upper panel, CP) or truncated HBV core promoters (Fig. 2B, upper panel, CPD1, CPD2 and CPD3), and their core promoter activities were examined in the presence or absence of TGF-β1. The reporter assay revealed that the intact HBV core promoter activity was significantly reduced by 53% after TGF-β1 treatment (Fig. 2B, lower panel, CP). Meanwhile, TGF-β1 showed similar repressive ability over the reporter CPD1, with 58% of the promoter activity corresponding to their TGF-β1 untreated control cells (Fig. 2B, lower panel, CPD1). Reporter with further deletion from the 5′-end of HBV core promoter showed severe reduction of the core reporter activity, of which the repressive abilities mediated by TGF-β1 treatment was significantly rescued as compared to its own mock control (Fig. 2B, lower panel, CPD2). This implies that the predominant element responsible for the inhibitory effect of TGF-β1 is likely to be embedded within the 5′-end of HBV core promoter, probably in the region between 1656 and 1675. Since the binding element of HNF-4α, a critical transcription factor for HBV pgRNA biosynthesis [10], [12], is located within this region, we proposed that HNF-4α might serve as the responsive factor in the suppression of HBV triggered by TGF-β1. To further confirm this speculation, several reporter plasmids with point mutations inside the two HNF-4α-binding elements (HNF4BEs), which have been reported to specifically disrupt the binding of HNF-4α to HBV core promoter [12], [26], were constructed (Fig. 2C, upper panel). Although the point mutation within either 5′- or 3′-HNF4BE (NEm or Npm) diminished HBV core promoter activity, the NEm and Npm mutant showed only moderate repression (68% and 66% of control cells, respectively) in their promoter activities after TGF-β1 treatment (Fig.2C, lower panel). A reporter with double mutations in both HNF4BEs (NEpm) showed complete resistance to the inhibitory effect of TGF-β1, even though the promoter activity of NEpm mutant was much weaker than that of wild-type core promoter (Fig. 2C, lower panel, NEpm). Our results suggested that intact HNF4BEs are essential for the down-regulation of HBV core promoter activity by TGF-β1 treatment.

### Intact HNF-4α binding sites within the HBV core promoter are essential for the antiviral effect of TGF-β1

**Figure 3:**
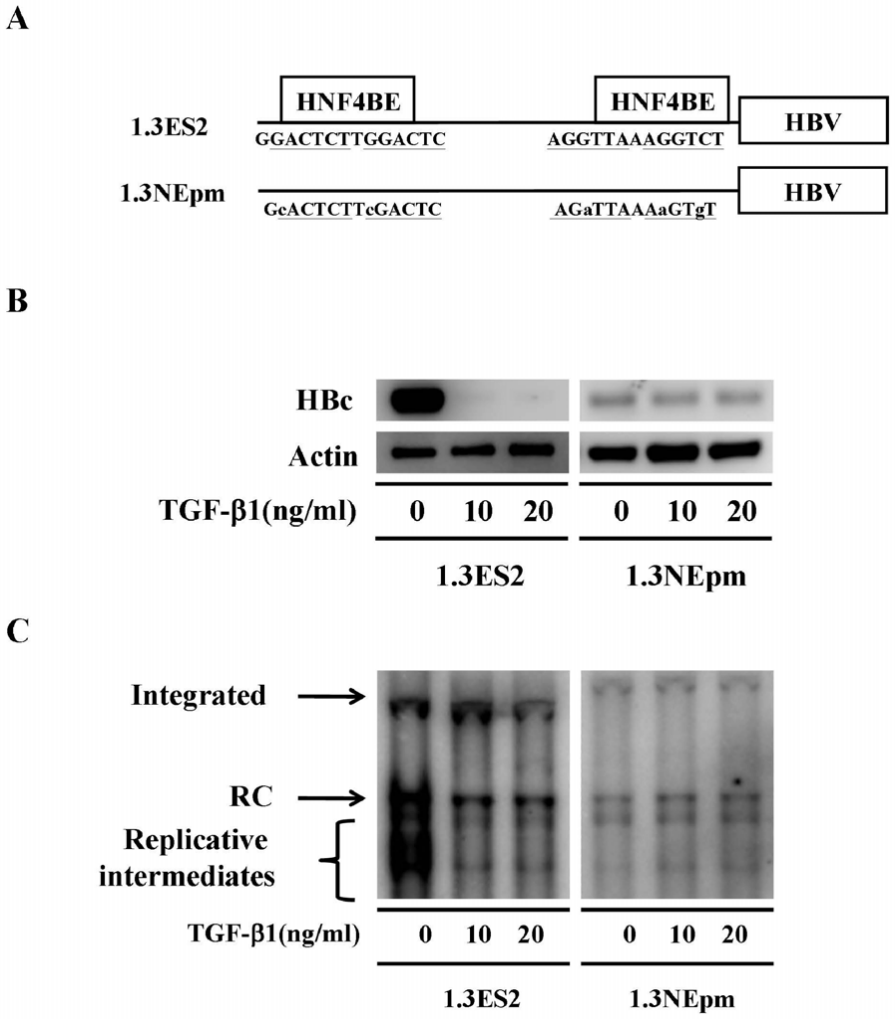
The HNF-4α binding sites within the HBV core promoter are essential elements for the TGF-β1-mediated suppression. (A) Schematic illustration of HBV-expressing plasmids with HNF-4α binding sites mutations within the HBV core promoter region. (B) Stably HBV-producing cell lines (1.3ES2 and 1.3NEpm) were treated with or without TGF-β1 for 6 days. To determine the expression level of HBc, cell lysate was extracted and subjected to Western blot analysis. The loaded amount of total protein was adjusted by the expression level of actin. (C) Total DNA was extracted from stable HBV-producing cell lines followed by HindIII digestion, and the viral replicative intermediates were examined by Southern blot analysis using an HBV-specific probe. The integrated HBV genomes were revealed as the internal control for equal amount of sample loading. Bands corresponding to the integrated genome (integrated), the relaxed circular double-stranded DNA (RC), the replicative intermediates are indicated individually.

Next, we examined whether the intact HNF4BEs within the HBV core promoter are essential for the inhibitory effect of TGF-β1 on HBV replication. To elucidate the role of HNF4BEs in TGF-β1-mediated HBV repression, an HNF4BE doubly-mutated HBV-producing cell line, 1.3NEpm, was established (Fig. 3A). As mentioned above, TGF-β1 treatment resulted in a substantial reduction of HBc expression in wild-type 1.3ES2 cells (Fig. 3B, 1.3ES2). Moreover, our data demonstrated that TGF-β1 treatment obviously repressed HBV replication as shown by the reduction of RC and viral replicative intermediates (Fig. 3C, 1.3ES2). With mutations in the HNF4BEs of HBV core promoter, 1.3NEpm cells produced relatively fewer amounts of HBc than wild-type 1.3ES2 cells did (Fig. 3B, 1.3NEpm). Interestingly, we found that TGF-β1 treatment failed to decrease the expression of HBc in 1.3NEpm cells as demonstrated by its relative low but consistently expressed HBc after TGF-β1 treatment (Fig. 3B, 1.3NEpm). Coincidently, the replication level of HBV in 1.3NEpm cells was relative less than that in 1.3ES2 cells. Meanwhile, we observed that TGF-β1 treatment failed to exert its inhibitory effects on the modulation of HBV replication in 1.3NEpm cells (Fig. 3C). Taken together, our data suggests that the binding of HNF-4α to the HBV core promoter is essential for the inhibitory effects of TGF-β1 on HBV replication.

### TGF-β1 suppresses HBV replication by manipulating HNF-4α expression

**Figure 4:**
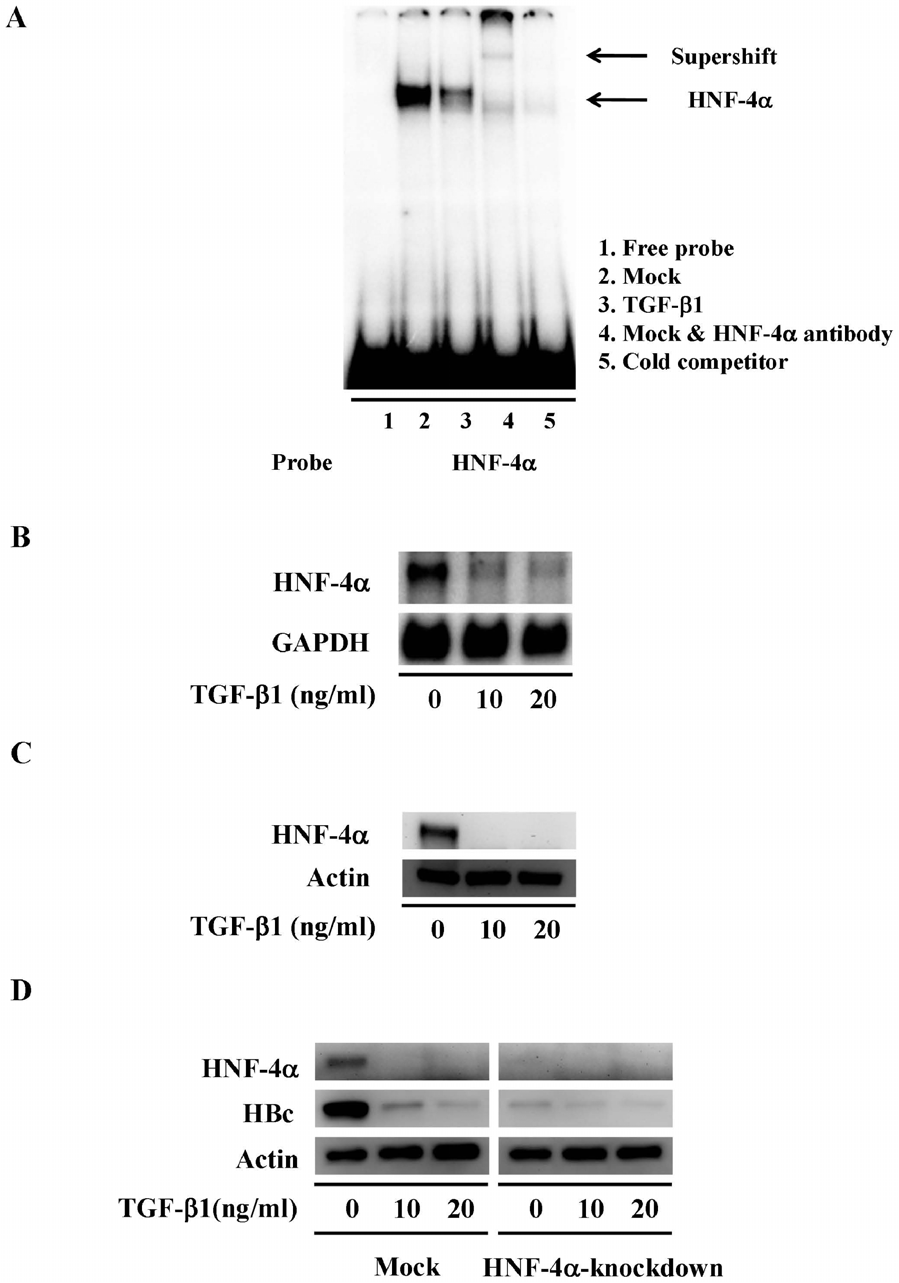
TGF-β1 treatment represses HNF-4α expression in HBV-producing cells. (A) The binding activity of endogenous HNF-4α in the presence or absence of TGF-β1 was examined by EMSA analysis. Nuclear extracts were prepared from HepG2 cells with or without TGF-β1 treatment for 2 days, and then incubated with HBV-specific probes containing HNF-4α binding element. Lane 1 is a free probe control without any nuclear extracts. Lane 2 is the specific probe incubated with HepG2 nuclear extracts. Lane 3 is the specific probe incubated with TGF-β1-treated nuclear extracts. Lane 4 is the specific probe incubated with HepG2 nuclear extracts and antibody against HNF-4α. Lane 5 is the specific probe incubated with HepG2 nuclear extracts and 100-fold molar excess cold competitor. (B) 1.3ES2 cells were plated followed by TGF-β1 treatment for 6 days. Total RNA was extracted analyzed by Northern blot with an HNF-4α specific probe. The expression of GAPDH was used as loading control. (C) 1.3ES2 cells were treated with TGF-β1 for 6 days. To examine the expression of HNF-4α, the cell lysate was extracted and subjected by Western blot analysis. The loaded amount of total protein was adjusted by the expression level of actin. (D) 1.3ES2 cells were infected with lentivirus carrying HNF-4α shRNA. The transduced cells were selected with puromycin, and then cells were treated by TGF-β1 for 6 days. The cell lysate was collected for Western blot analysis to determine the expression level of HNF-4α and HBc. The total protein loaded was adjusted by actin expression level.

We have provided evidence that the intact HNF4BEs within the HBV core promoter play an important role in TGF-β1-mediated antiviral activity. The subsequent question of interest is how TGF-β1 manipulates the binding of HNF-4α to the HBV core promoter. To confirm whether TGF-β1 treatment interferes with the interaction between HNF-4α and its responsive element, the binding activity of endogenous HNF-4α in the presence or absence of TGF-β1 was examined by EMSA analysis (Fig. 4A). An EMSA probe corresponding to the 5′-HNF4BE located within HBV core promoter was chose to perform EMSA analysis. The binding activity of endogenous HNF-4α to its responsive probe was significantly reduced after TGF-β1 treatment (Fig. 4A). We next investigated whether TGF-β1 affects the expression of HNF-4α in 1.3ES2 cells. Surprisingly, we found that TGF-β1 treatment resulted in a dramatic reduction in HNF-4α mRNA (Fig. 4B). Furthermore, a significant disappearance of HNF-4α protein by TGF-β1 treatment was observed by Western blot analysis (Fig. 4C).

### HNF-4α plays a crucial role in the suppressive effect of TGF-β1 on HBV replication

**Figure 5:**
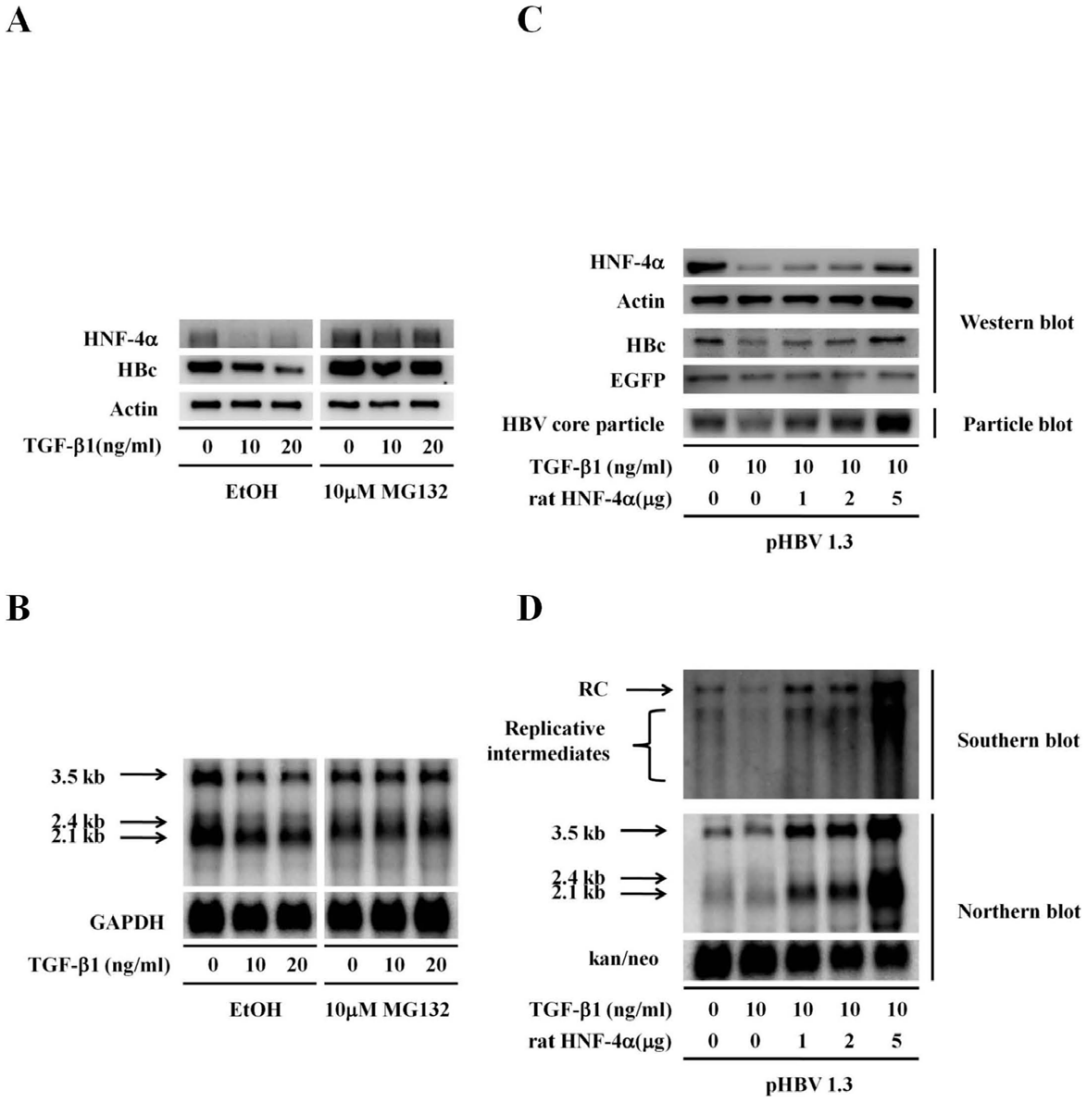
HNF-4α plays a crucial role in the TGF-β1-associated inhibitory effect on HBV replication. 1.3ES2 cells were treated with or without 10 µM MG132 for 1 hour and TGF-β1 for 12 hours. (A) The cell lysate was collected for Western blot analysis for the detection of HNF-4α and HBc. The loading amount of total protein was monitored by actin. (B) Total RNA was extracted, and the expression of HBV transcripts were revealed by Northern blot with using an HBV-specific probe. The expression of GAPDH was used as the loading control. (C) HepG2 cells were transfected with pHBV1.3 plasmids and an increased amount of rat HNF-4α-expressing plasmids. After treated with TGF-β1 for 2 days, the cell lysate was collected for the detection of HNF-4α and HBc by Western blot analysis. The expression of core particles was examined by particle blot analysis. The equal amount of total protein was revealed by the level of actin. The amount of HBc and HBV particle were also adjusted by the expression level of EGFP for monitoring the transfection efficiency. (D) Total RNA was extracted, and the expression of HBV transcripts were revealed by Northern blot with an HBV-specific probe. The expression of the kanamycin-neomycin phosphate transferase (kan/neo) was used as the loading control. Total DNA was extracted followed by HindIII digestion. The viral replicative intermediates were examined by Southern blot analysis using an HBV-specific probe. Bands corresponding to the relaxed circular double-stranded DNA (RC), the replicative intermediates are indicated individually.

To confirm the significance of HNF-4α protein in mediating the antiviral effect of TGF-β1, the expression level of endogenous HNF-4α was specifically reduced by RNA interference (RNAi) technique, and then the inhibitory effects of TGF-β1 were analyzed. Consistent with the previous study [27], the reduction of HNF-4α expression by RNAi was sufficient to trigger HBc repression even without TGF-β1 treatment (Fig. 4D). Although TGF-β1 significantly repressed HBc expression in these mock control cells, the level of TGF-β1-mediated HBc reduction in these HNF-4α knock-down cells was relatively less significant (Fig. 4D). Since the metabolism of HNF-4α by TGF-β1 treatment has been reported to be mediated through the proteasome-dependent degradation pathway [28], we next examined whether blocking the TGF-β1-mediated HNF-4α degradation by proteasome inhibitor MG132 could alter the consequence of HBc suppression by TGF-β1 treatment. We found that MG132 treatment not only protected HNF-4α protein from degradation but also prevented HBc from TGF-β1-mediated repression (Fig. 5A). Interestingly, Northern blot analysis revealed that the reduction of the 3.5 kb transcripts by TGF-β1 was also prohibited while cells were pretreated with MG132 (Fig. 5B). Taken together, our data indicate that the manipulation of HNF-4α expression plays a crucial role in diminishing of HBc expression as well as pgRNA transcription during TGF-β1 treatment.

### Recovery of HNF-4α expression in TGF-β1-treated cells can rescue HBV replication

To further confirm that whether the expression level of HNF-4α is the key parameter in modulating of the HBV replication by TGF-β1, HBV-producing cells were induced to express different amounts of rat HNF-4α in the presence of TGF-β1. Our data revealed that ectopic expression of rat HNF-4α alone was sufficient to rescue the impaired HBc expression caused by TGF-β1 treatment (Fig. 5C, Western blot), and that the assembling of intracellular viral particles could also be restored by rat HNF-4α overexpression (Fig. 5C, Particle blot). Moreover, the expression level of HBV transcripts (such as 3.5 kb pre-C mRNA/pgRNA and 2.4–2.1 kb subgenomic mRNAs) and intracellular viral replicative intermediates were both elevated in parallel with the increased amounts of rat HNF4 (Fig. 5D, Northern blot and Southern blot). In conclusion, we provide evidence to support our scenario in which the crucial hepatic transcription factor HNF-4α is indispensable for the suppressive effect of TGF-β1 on HBV replication.

## Discussion

### TGF-β1 inhibits HBV replication through the modulation of HNF-4α

**Figure 6:**
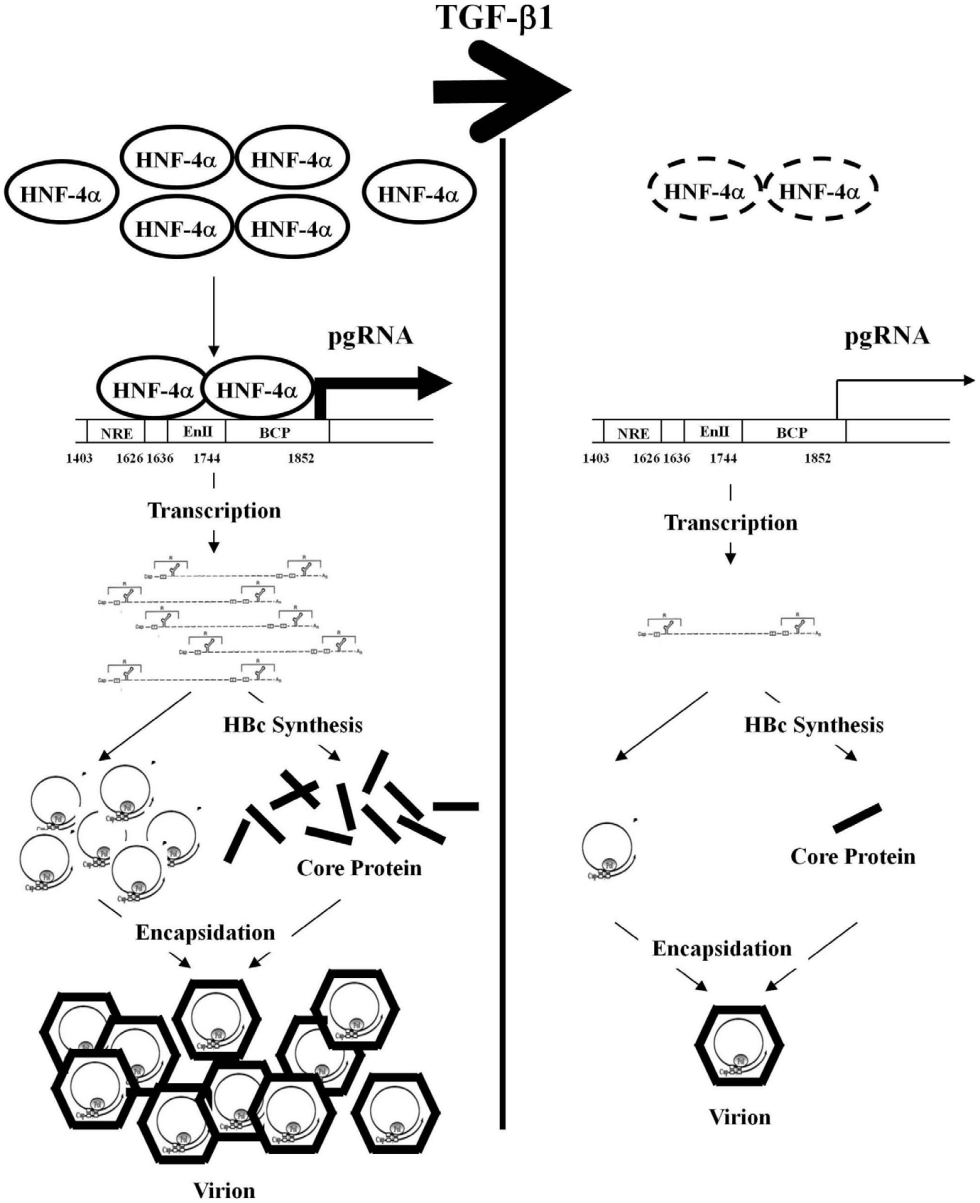
Schematic illustration of our model in which TGF-β1 exerts its anti-HBV effect through the modulation of cellular HNF-4α expression level. In the hepatocytes, the abundantly expressed HNF-4α efficiently activates the transcription of HBV pgRNA. The accumulated pgRNA not only serve as the templates for synthesis of viral core protein but also assemble with HBV core protein to form viral nucleocapsids. In TGF-β1-treated hepatocytes, the expression level of HNF-4α is dramatically reduced, which severely interfere with the synthesis of pgRNA and HBV core protein. Lacking of those viral components, the formation of nucleocapsid and HBV replication are substantially suppressed.

Our results indicate that TGF-β1 exerts its anti-HBV effect through the modulation of cellular HNF-4α as explained in a proposed model (Fig. 6). Briefly, the abundantly expressed HNF-4α is loaded to the HNF4BE(s) within HBV core promoter and subsequently enhances the transcription of HBV pgRNA. The pgRNA serves as the mRNA for HBc synthesis, and then these HBc in turn assemble with pgRNA to form viral particle. In the presence of TGF-β1, the expression of HNF-4α is diminished. Lacking of HNF-4α, the HBc synthesis is dramatically reduced, and subsequently the formation of nucleocapsid as well as HBV replication is repressed. Our study suggests that liver-enriched transcription factor HNF-4α plays vital role in HBV suppression by TGF-β1.

### The inhibitory effects of TGF-β1 on the modulation of HBV transcription

In this report, we continued our previous research on TGF-β1-mediated HBV repression and explored the mechanism how TGF-β1 interferes with HBV replication. Our data suggested that the reduction of cellular HNF-4α by TGF-β1 treatment dramatically diminishes the expression level of HBV pgRNA, but not pre-C mRNA. Although the promoter regions of pgRNA and pre-C mRNA are highly overlapped and share the same transcription factor binding motifs, it has been indicated that the transcriptions of the pgRNA and pre-C mRNA are differentially regulated and are directed by two distinct promoters [8]. The basal elements of these two promoters are similar but genetically separable, with each consisting of its own transcriptional initiator and a TATA box-like sequence [8]. Furthermore, it has been reported that HNF-4 differentially regulates pgRNA and pre-C mRNA, of which HNF-4 specifically activates the transcription of pgRNA, but not pre-C mRNA [8], [12]. One possible explanation was provided by Yu et. al., they found that the 3′ HNF-4 binding element was overlapped with the TATA-box of pre-C promoter, and the binding of HNF-4 on this region might interfere with the assembling of the initial transcription complex for pre-C mRNA production [8]. As a consequence, it is not surprising that the reduction of HNF-4α by TGF-β1 has more impact on the suppression of pgRNA than pre-C mRNA.

It has been well demonstrated that HNF-4α binding sites located within HBV core promoter (enhancer II) region and enhancer I/X promoter region are critical cis-acting elements for the regulation of HBV replication [29], [30]. However, TGF-β1 differentially regulates HBV core promoter and enhancer I/X promoter, that is, TGF-β1 significantly inhibited HBV core promoter but only slightly modulates enhancer I/X promoter. It is likely due to that HNF-4α has much higher binding affinity for its responsive element(s) in HBV core promoter (enhancer II) than that in enhancer I/X promoter [31].

In addition to 3.5 Kb HBV transcripts, we found that the expressions of 2.1–2.4 Kb HBV subgenomic RNAs were also repressed by TGF-β1 treatment (Fig. 1B). Several transcription factors (including HNF1, HNF3, SP1, TBP, CBF, NF-Y and NF1), but not HNF-4, have been suggested to locate onto HBV surface promoter and regulate the expression of these HBV subgenomic RNAs [4]. Among them, the expression of HNF-3β has been proved to substantially increase the transcriptional activity of surface promoter [32]. Interestingly, our preliminary data showed that the reduction of 2.1–2.4 Kb HBV transcripts were in parallel with the repression of HNF-3β expression during TGF-β1 treatment (data not shown). Considering the hierarchical role of HNF-4α, it raised the possibility that the changes in HNF-4α expression level might lead to the alteration of HNF-3β expression. This idea is supported by our previous research in which we demonstrated that overexpression of HNF-4α significantly upregulates the transcription of HNF-3β [33]. Moreover, we showed that overexpression of HNF-4α could rescue the expression of 2.1–2.4 Kb HBV transcripts under TGF-β1 treatment (Fig. 5D). Taken together, we believe that TGF-β1 might indirectly repressed HNF-3β expression, probably through reducing of HNF-4α expression, and consequently inhibited the expression of these HBV subgenomic RNAs.

### The role of HNF-4α in cytokine-mediated HBV clearance

The time course studies of viral DNA disappearance and T cells infiltration suggested that HBV clearance occurs before the destruction of infected hepatocytes [34], which indicates that cytokine-mediated non-cytopathic viral clearance plays an important role in the inhibition of HBV replication. Several cytokines including interleukin-4 (IL-4), IL-6, IL-18, interferons (IFNs), tumor necrosis factor-alpha (TNF-α) and TGF-β1 were demonstrated to effectively and noncytopathically suppress HBV replication [23], [35], [36], [37], [38], [39], [40]. Among them, TGF-β1 is the only cytokine reported to efficiently suppress HBV replication at a physiological concentration. In this report, we continue our previous study and clarify the detail mechanism by which TGF-β1 inhibits HBV replication. We found that TGF-β1 represses HBV replication primarily through the specifically inhibiting of viral core protein expression. This inhibitory effect triggered by TGF-β1 is similar to that caused by IL-4, IL-6, IFN-α, IFN-γ, or TNF-α, all of which have been reported to reduce HBV core protein level [38], [39], [40], [41]. However, the molecular mechanisms by which these cytokines exert their inhibitory effects on the core promoter are not well investigated. Here, we suggested that HNF-4α plays a crucial role in TGF-β1-mediated suppression of the HBV core promoter, which subsequently blocks HBc synthesis and HBV replication. Previous study of HBV infection in primary human liver cells suggested that Kupffer cells can secrete IL-6 upon viral recognition, and in turn inhibits HBV replication by downregulation of HNF-1α and HNF-4α [40]. Moreover, the study of a natural helioxanthin analogue showed that the main antiviral mechanism triggered by this compound is also mediated by downregulation of HNF-3 and HNF-4α [42]. Meanwhile, Scutellariae radix, one major component of traditional Chinese medicine Xiao-Chai-Hu-Tang, was suggested to suppress HBV production by compromising the binding between HNF-4α and HBV core promoter [43]. In conclusion, HNF-4α is certainly one of the critical targets involved in viral clearance during the modulation of HBV replication.

### Down-regulation of HNF-4α by TGF-β1 treatment

The repression of HNF-4α expression by TGF-β1 has been reported to be exerted through several distinct mechanisms [28], [44], [45]. For example, TGF-β1 induced the expression of transcription repressor Snail in hepatocytes, which in turn reduced the transcription of HNF-4α gene through direct binding of Snail within the HNF-4α promoter region [44]. In addition, another transcription repressor HMGA2 was activated through TGF-β1-induced Smad3 signaling pathway in mammary epithelial cells and subsequently suppressed the transcription of HNF-4α [45]. Moreover, TGF-β1 induces the elevation of miR-24 expression [46], which in turn targets to the 3′-UTR of HNF-4α and regulates the level of HNF-4α by a post-transcriptional control [47]. It is also suggested that the post-translational modification is involved in the suppression of HNF-4α activity, in which the phosphorylated HNF-4α prevents protein dimer formation and affects its own protein stability [48]. Besides, IL-1β and TGF-β1 are both reported to induce HNF-4α degradation via the proteasome dependant pathway, and the reduction of HNF-4α could be rescued by the addition of the proteasome inhibitor, MG132 [28], [49]. In our study, the transcriptional repression of HNF-4α was observed in the presence of TGF-β1 (Fig. 4B). Furthermore, we also observed that HNF-4α protein was repressed after TGF-β1 treatment (Fig. 4C), and the TGF-β1-induced HNF-4α degradation was prevented by the pretreatment of MG132 (Fig. 5A). A putative PEST signal has been predicted near the C-terminus of HNF-4α amino acid sequence [49], which might be responsible for the target of HNF-4α to rapid destruction and confer its instability after TGF-β1 treatment [50].

### HNF-4α plays a hierarchical role in hepatocyte differentiation and controls viral replication

HBV replication is not only regulated by the expression of hepatic transcription factors [25], but is also closely related to the differentiation status of hepatocytes [51]. Several lines of evidence suggest that modulating the expression of hepatic transcription factors is tightly linked to hepatocyte differentiation status. For example, the differentiated primary hepatocytes induced by dimethyl sulfoxide treatment reveal higher levels of HNF-1α, HNF-1β, HNF-3γ, and HNF-4α than proliferating cells [52]. A complicated cross-regulatory network between hepatic transcription factors has been constructed by analyzing the expression levels of transcription factors in developing liver [53]. Within this network, a core group of six hepatic transcription factors (HNF-1α, HNF-1β, HNF-3β, HNF-4α, HNF-6, and liver receptor homolog-1) are suggested to regulate with each other and the downstream hepatic regulators [53]. Of these, HNF-4α has been identified as the central regulator of multiple genes contributing to hepatocyte differentiation, including HNF-1α and pregnane X receptor [14], [54]. In this report, we demonstrated that TGF-β1 strongly represses the expression of HNF-4α, which raises the possibility that the loss of HNF-4α might consequently affect the differentiation status of hepatocytes. In addition to HNF-4α, we observed a significant reduction in the binding activity of HNF-1 and HNF-3 in our EMSA analysis after TGF-β1 treatment (data not shown). We also found that the expression level of HNF-1 and albumin were down-regulated by TGF-β1 treatment (data not shown). These findings imply that TGF-β1 is able to alter the expression of several transcription factors and the differentiation status of hepatocytes. Thus, we suggested that TGF-β1 diminishes the expression of HNF-4α, which may regulate HBV replication not only by directly reducing HBV core protein biosynthesis but also by indirectly affecting other hepatic genes and hepatocyte differentiation status.

### The immunosuppressive roles of TGF-β1 in HBV infection

It is well established that TGF-β1 functions as a negative regulator of cellular and humoral immune responses during HBV infection, including lymphocyte proliferation, IFN-γ secretion, and antibody production [55]. Recently, studies in chronically HBV-infected patients revealed that the elevation of serum TGF-β1 is accompanied by an increased frequency of regulatory T cells (Tregs) [56]. Moreover, it has been well demonstrated that TGF-β1 converts native T cells into Trgs in the periphery [57]. It provides new insight into the immunosuppressive role of TGF-β1 in viral hepatitis. Growing evidence suggests that Tregs control the immune responses to hepatitis viruses by modulating effector T cell activation [58], [59]. During chronic virus infection, Tregs appear to restrain overactive immune responses and eliminate host tissue damage [60]. Furthermore, increased numbers of cytotoxic T lymphocyte are observed after the Tregs decline, supporting a role for Tregs in controlling immune responses during HBV infection [61]. In this study, we have shown clearly that TGF-β1 can efficiently repress HBV replication by impairing HNF-4α expression, which likely contributes to the restriction of virus replication during HBV infection. Taken together, all the evidence suggests that the elevated TGF-β1 in chronically HBV-infected patients might have dual biological significance, both reducing viral DNA load and preventing liver damage during persistent HBV infection. With virus-suppressing and immune-restraining features, we believe that TGF-β1 might play a unique role in cytokine-mediated HBV clearance. However, the possible therapeutic applications of TGF-β1 in HBV patients need to be further investigated.

## Materials and Methods

### Cell Culture and Transfection

The stable HBV-producing cell line, 1.3ES2 cells, is a clone derivative of HepG2 cells in which the 1.3 copies of the entire HBV genome was stably integrated [62]. Human hepatoblastoma cell line, HepG2 cells, and the stably HBV-producing cell line, 1.3ES2 cells, were maintained as previously described [23]. HepG2 cells were transfected with HBV-expressing plasmids or reporter plasmids by using Arrest-In transfection reagent (Thermo Scientific, Waltham, MA). HNF4BEs-mutated HBV-producing cell line, 1.3NEpm, was established by transfected with HBV HNF4BEs-mutated plasmid and selected with hygromycin. To assess the antiviral effect of TGF-β1 on HBV replication, cells were treated with 10 ng/ml or 20 ng/ml of TGF-β1 (R&D Systems, Minneapolis, MN).

### Plasmid Construction

To analyze HBV core promoter activity, several reporter plasmids were constructed with the luciferase gene under the control of distinct core promoter regions and were transfected into HepG2 cells to analyze their luciferase activity. The HBV core promoter (CP: nucleotides 1636–1851) was amplified from the ayw subtype of the HBV genome [63] and was subcloned between MluI and HindIII restriction sites on the pGL3-Basic luciferase vector (Promega Corporation, Madison, WI). Reporters with with the HBV enhancer I/X promoter (EnI/X: nucleotides 947–1372) and HBV core promoter deletions (CPD1: nucleotides 1656–1851, CPD2: nucleotides 1675–1851, and CPD3: nucleotides 1708–1851) were also constructed by the same strategy. HNF4BE mutants were generated using the QuikChange II Site-Directed Mutagenesis Kit (Stratagene, La Jolla, CA) to alter the parental HNF-4α binding sequences in either the HBV core promoter reporter or HBV-expressing plasmids (pHBV1.3), which was constructed as previously described [62]. The mutant of the 5′-HNF4BE (NEm) was constructed by substituting the wild-type HNF4BE (GGACTCTTGGACTC) with a mutant 5′-HNF4BE (GcACTCTTcGACTC), which was not bound by HNF-4α [12]. The mutant 3′-HNF4BE (Npm) was generated by replacing the wild-type 3′-HNF4BE (AGGTTAAAGGTCT) with the mutant 3′-HNF4BE (AGaTTAAAaGTgT), which impaired the interaction with HNF-4α [26]. The construct with mutations at both 5′- and 3′-HNF4BEs was named as NEpm. In this report, the unique EcoRI recognition site within the HBV genome is defined as nucleotide 1.

### Northern Blot Analysis

Total RNA was isolated using TRIzol solution (Invitrogen, Carlsbad, CA) and was separated on a 1.2% formaldehyde-agarose gel and transferred onto positively charged Hybond-N+ nylon membranes (GE Healthcare, Piscataway, NJ). The membranes were UV cross-linked and hybridized with ^32^P-labeled HBV-specific or HNF-4α-specific probes, which were generated by a Prime-a-Gene Labeling System (Promega Corporation, Madison, WI). The loading amount of total RNA was monitored using a GAPDH-specific probe. The isotope signals were detected with a phosphoimager FLA-2000 image system (Fujifilm, Minato-ku, Tokyo, Japan).

### Primer Extension Analysis

Total RNA was isolated, and the same amount total RNA was applied for the relative quantitation of HBV pregenomic RNA (pgRNA) and pre-C mRNA as previously described by Ou et al. [64]. The abundance of pre-C mRNA and pgRNA was detected by a ^32^P-labeled 28-mer primer corresponding to HBV nucleotides 2051–2024 [64].

### Western Blot Analysis

Total protein was separated by 12% sodium dodecyl sulfate-polyacrylamide gel electrophoresis (SDS-PAGE) and transferred onto polyvinylidene fluoride membranes (Millipore, Billerica, MA). The membranes were blocked with 5% nonfat milk and incubated with HBc-specific or HNF-4α-specific antibodies. The immunoblot signals were examined using enhanced chemiluminescence reagent (Millipore, Billerica, MA) and detected with a UVP BioSpectrum 500 image system (UVP, Upland, CA). Antibody against HBc was purchased from Dako (Carpinteria, CA), anti-HNF-4α antibody from Santa Cruz Biotechnology (Santa Cruz, CA), anti-actin antibody from Millipore (Millipore, Billerica, MA) and anti-EGFP antibody from Clontech (Clontech, Mountain View, CA).

### Particle Blot Analysis

Analysis of intracellular HBV core particles was performed as previously described [65], [66]. In brief, cell lysate was separated on a 1.2% native agarose gel and transferred onto polyvinylidene fluoride membranes or nylon membranes for the detection of HBV core particles. HBV core particles were examined by immunoblot analysis using an anti-HBc antibody. Capsid-associated nucleic acids were released from the core particles in situ by denaturing the membranes with 0.2 N NaOH/1.5 M NaCl, and neutralizing with 0.2 N Tris-HCl/1.5 M NaCl. Finally, the membranes were hybridized with an HBV-specific probe as previously described [23].

### Luciferase Assay

To analyze the activities of HBV core promoters, HepG2 cells were transfected with each reporter construct and CMV promoter-driven beta-galactosidase plasmid for monitoring the transfection efficiency. One day after cotransfection, cells were treated with or without TGF-β1 (10 ng/ml) for two days. Luciferase and beta-galactosidase activities were quantified with Bright Glo luciferase assay kits (Promega Corporation, Madison, WI) and Beta Glo luciferase assay kits (Promega Corporation, Madison, WI), respectively. The luciferase signals were determined using a Hidex Chameleon luminescence reader (Hidex, Turku, Finland).

### Electrophoretic Mobility Shift Assay (EMSA)

EMSA was performed as previously described by Niehof et al. [67]. In brief, nuclear extracts were prepared from HepG2 cells treated with or without TGF-β1 for two days by nuclear and cytoplasmic extraction reagents NE-PER (Thermo Scientific, Waltham, MA). The oligonucleotides were purchased with the following sequences: AGAGGACTCTTGGACTCTCAGCA and TGCTGAGAGTCCAAGAGTCCTCTT, annealed, and 5′-end-labeled with ^32^P by T4 polynucleotide kinase (Promega Corporation, Madison, WI). The oligonucleotide were then extracted by phenol/chloroform and purified by Sephadex G-25 column (GE Healthcare, Piscataway, NJ). Super shift assays were done with HNF-4α specific antibody (sc-6556) was purchased from Santa Cruz Biotechnology (Santa Cruz, CA). For competition EMSA, 100 fold excess cold oligonucleotides were used.

### RNA Interference

Lentivirus carrying HNF-4α-specific short hairpin (sh)RNA (purchased from the National RNAi Core Facility, Taiwan) was used to knock down endogenous HNF-4α. The target sequence within the HNF-4α gene was CGAGCAGATCCAGTTCATCAA. Stably HBV-producing 1.3ES2 cells were spin infected with lentivirus in 8 µg/ml polybrene at 1,100× *g* for 30 min (multiplicity of infection = 2), and the media was refreshed one day after the infection. The lentivirus-infected 1.3ES2 cells were then selected with 2 µg/ml of puromycin for two days and followed by TGF-β1 treatment as described above.

## References

[1] Chen CJ, Yang HI, Su J, Jen CL, You SL (2006). Risk of hepatocellular carcinoma across a biological gradient of serum hepatitis B virus DNA level.. JAMA.

[2] Farazi PA, DePinho RA (2006). Hepatocellular carcinoma pathogenesis: from genes to environment.. Nat Rev Cancer.

[3] Seeger C, Mason WS (2000). Hepatitis B virus biology.. Microbiol Mol Biol Rev.

[4] Moolla N, Kew M, Arbuthnot P (2002). Regulatory elements of hepatitis B virus transcription.. J Viral Hepat.

[5] Tang H, Banks KE, Anderson AL, McLachlan A (2001). Hepatitis B virus transcription and replication.. Drug News Perspect.

[6] Yaginuma K, Koike K (1989). Identification of a promoter region for 3.6-kilobase mRNA of hepatitis B virus and specific cellular binding protein.. J Virol.

[7] Yuh CH, Chang YL, Ting LP (1992). Transcriptional regulation of precore and pregenomic RNAs of hepatitis B virus.. J Virol.

[8] Yu X, Mertz JE (1996). Promoters for synthesis of the pre-C and pregenomic mRNAs of human hepatitis B virus are genetically distinct and differentially regulated.. J Virol.

[9] Li J, Ou JH (2001). Differential regulation of hepatitis B virus gene expression by the Sp1 transcription factor.. J Virol.

[10] Raney AK, Johnson JL, Palmer CN, McLachlan A (1997). Members of the nuclear receptor superfamily regulate transcription from the hepatitis B virus nucleocapsid promoter.. J Virol.

[11] Tang H, McLachlan A (2001). Transcriptional regulation of hepatitis B virus by nuclear hormone receptors is a critical determinant of viral tropism.. Proc Natl Acad Sci U S A.

[12] Yu X, Mertz JE (2003). Distinct modes of regulation of transcription of hepatitis B virus by the nuclear receptors HNF4alpha and COUP-TF1.. J Virol.

[13] Kuo CJ, Conley PB, Chen L, Sladek FM, Darnell JE (1992). A transcriptional hierarchy involved in mammalian cell-type specification.. Nature.

[14] Li J, Ning G, Duncan SA (2000). Mammalian hepatocyte differentiation requires the transcription factor HNF-4alpha.. Genes Dev.

[15] Odom DT, Zizlsperger N, Gordon DB, Bell GW, Rinaldi NJ (2004). Control of pancreas and liver gene expression by HNF transcription factors.. Science.

[16] Parviz F, Matullo C, Garrison WD, Savatski L, Adamson JW (2003). Hepatocyte nuclear factor 4alpha controls the development of a hepatic epithelium and liver morphogenesis.. Nat Genet.

[17] Zhang H, Ozaki I, Mizuta T, Hamajima H, Yasutake T (2006). Involvement of programmed cell death 4 in transforming growth factor-beta1-induced apoptosis in human hepatocellular carcinoma.. Oncogene.

[18] Bissell DM, Wang SS, Jarnagin WR, Roll FJ (1995). Cell-specific expression of transforming growth factor-beta in rat liver. Evidence for autocrine regulation of hepatocyte proliferation.. J Clin Invest.

[19] Martin-Vilchez S, Sanz-Cameno P, Rodriguez-Munoz Y, Majano PL, Molina-Jimenez F (2008). The hepatitis B virus X protein induces paracrine activation of human hepatic stellate cells.. Hepatology.

[20] Murawaki Y, Nishimura Y, Ikuta Y, Idobe Y, Kitamura Y (1998). Plasma transforming growth factor-beta 1 concentrations in patients with chronic viral hepatitis.. J Gastroenterol Hepatol.

[21] Akpolat N, Yahsi S, Godekmerdan A, Demirbag K, Yalniz M (2005). Relationship between serum cytokine levels and histopathological changes of liver in patients with hepatitis B.. World J Gastroenterol.

[22] Shirai Y, Kawata S, Tamura S, Ito N, Tsushima H (1994). Plasma transforming growth factor-beta 1 in patients with hepatocellular carcinoma. Comparison with chronic liver diseases.. Cancer.

[23] Chou YC, Chen ML, Hu CP, Chen YL, Chong CL (2007). Transforming growth factor-beta1 suppresses hepatitis B virus replication primarily through transcriptional inhibition of pregenomic RNA.. Hepatology.

[24] Bock CT, Malek NP, Tillmann HL, Manns MP, Trautwein C (2000). The enhancer I core region contributes to the replication level of hepatitis B virus in vivo and in vitro.. J Virol.

[25] Quasdorff M, Protzer U (2010). Control of hepatitis B virus at the level of transcription.. J Viral Hepat.

[26] Yu X, Mertz JE (2001). Critical roles of nuclear receptor response elements in replication of hepatitis B virus.. J Virol.

[27] Cheng YC, Liang CM, Chen YP, Tsai IH, Kuo CC (2009). F-spondin plays a critical role in murine neuroblastoma survival by maintaining IL-6 expression.. J Neurochem.

[28] Lucas Sd S, Lopez-Alcorocho JM, Bartolome J, Carreno V (2004). Nitric oxide and TGF-beta1 inhibit HNF-4alpha function in HEPG2 cells.. Biochem Biophys Res Commun.

[29] Zheng Y, Li J, Ou JH (2004). Regulation of hepatitis B virus core promoter by transcription factors HNF1 and HNF4 and the viral X protein.. J Virol.

[30] Garcia AD, Ostapchuk P, Hearing P (1993). Functional interaction of nuclear factors EF-C, HNF-4, and RXR alpha with hepatitis B virus enhancer I.. J Virol.

[31] Yu X, Mertz JE (1997). Differential regulation of the pre-C and pregenomic promoters of human hepatitis B virus by members of the nuclear receptor superfamily.. J Virol.

[32] Raney AK, Zhang P, McLachlan A (1995). Regulation of transcription from the hepatitis B virus large surface antigen promoter by hepatocyte nuclear factor 3.. J Virol.

[33] Chen ML, Lee KD, Huang HC, Tsai YL, Wu YC (2010). HNF-4alpha determines hepatic differentiation of human mesenchymal stem cells from bone marrow.. World J Gastroenterol.

[34] Guidotti LG, Rochford R, Chung J, Shapiro M, Purcell R (1999). Viral clearance without destruction of infected cells during acute HBV infection.. Science.

[35] Kimura K, Kakimi K, Wieland S, Guidotti LG, Chisari FV (2002). Interleukin-18 inhibits hepatitis B virus replication in the livers of transgenic mice.. J Virol.

[36] McClary H, Koch R, Chisari FV, Guidotti LG (2000). Relative sensitivity of hepatitis B virus and other hepatotropic viruses to the antiviral effects of cytokines.. J Virol.

[37] Guidotti LG, Morris A, Mendez H, Koch R, Silverman RH (2002). Interferon-regulated pathways that control hepatitis B virus replication in transgenic mice.. J Virol.

[38] Lin SJ, Shu PY, Chang C, Ng AK, Hu CP (2003). IL-4 suppresses the expression and the replication of hepatitis B virus in the hepatocellular carcinoma cell line Hep3B.. J Immunol.

[39] Kuo TM, Hu CP, Chen YL, Hong MH, Jeng KS (2009). HBV replication is significantly reduced by IL-6.. J Biomed Sci.

[40] Hosel M, Quasdorff M, Wiegmann K, Webb D, Zedler U (2009). Not interferon, but interleukin-6 controls early gene expression in hepatitis B virus infection.. Hepatology.

[41] Romero R, Lavine JE (1996). Cytokine inhibition of the hepatitis B virus core promoter.. Hepatology.

[42] Ying C, Li Y, Leung CH, Robek MD, Cheng YC (2007). Unique antiviral mechanism discovered in anti-hepatitis B virus research with a natural product analogue.. Proc Natl Acad Sci U S A.

[43] Tseng YP, Wu YC, Leu YL, Yeh SF, Chou CK (2010). Scutellariae radix suppresses hepatitis B virus production in human hepatoma cells.. Front Biosci (Elite Ed).

[44] Cicchini C, Filippini D, Coen S, Marchetti A, Cavallari C (2006). Snail controls differentiation of hepatocytes by repressing HNF4alpha expression.. J Cell Physiol.

[45] Ishikawa F, Nose K, Shibanuma M (2008). Downregulation of hepatocyte nuclear factor-4alpha and its role in regulation of gene expression by TGF-beta in mammary epithelial cells.. Exp Cell Res.

[46] Huang S, He X, Ding J, Liang L, Zhao Y (2008). Upregulation of miR-23a approximately 27a approximately 24 decreases transforming growth factor-beta-induced tumor-suppressive activities in human hepatocellular carcinoma cells.. Int J Cancer.

[47] Takagi S, Nakajima M, Kida K, Yamaura Y, Fukami T (2009). MicroRNAs regulate human hepatocyte nuclear factor 4alpha, modulating the expression of metabolic enzymes and cell cycle.. J Biol Chem.

[48] Hong YH, Varanasi US, Yang W, Leff T (2003). AMP-activated protein kinase regulates HNF4alpha transcriptional activity by inhibiting dimer formation and decreasing protein stability.. J Biol Chem.

[49] Wang B, Cai SR, Gao C, Sladek FM, Ponder KP (2001). Lipopolysaccharide results in a marked decrease in hepatocyte nuclear factor 4 alpha in rat liver.. Hepatology.

[50] Rechsteiner M, Rogers SW (1996). PEST sequences and regulation by proteolysis.. Trends Biochem Sci.

[51] Le Jossic C, Glaise D, Corcos L, Diot C, Dezier JF (1996). trans-Acting factors, detoxication enzymes and hepatitis B virus replication in a novel set of human hepatoma cell lines.. Eur J Biochem.

[52] Mizuguchi T, Mitaka T, Hirata K, Oda H, Mochizuki Y (1998). Alteration of expression of liver-enriched transcription factors in the transition between growth and differentiation of primary cultured rat hepatocytes.. J Cell Physiol.

[53] Kyrmizi I, Hatzis P, Katrakili N, Tronche F, Gonzalez FJ (2006). Plasticity and expanding complexity of the hepatic transcription factor network during liver development.. Genes Dev.

[54] Watt AJ, Garrison WD, Duncan SA (2003). HNF4: a central regulator of hepatocyte differentiation and function.. Hepatology.

[55] Kakumu S, Ito Y, Takayanagi M, Yoshioka K, Wakita T (1993). Effect of recombinant human transforming growth factor beta 1 on immune responses in patients with chronic hepatitis B.. Liver.

[56] Yang G, Liu A, Xie Q, Guo TB, Wan B (2007). Association of CD4+CD25+Foxp3+ regulatory T cells with chronic activity and viral clearance in patients with hepatitis B.. Int Immunol.

[57] Chen W, Jin W, Hardegen N, Lei KJ, Li L (2003). Conversion of peripheral CD4+CD25− naive T cells to CD4+CD25+ regulatory T cells by TGF-beta induction of transcription factor Foxp3.. J Exp Med.

[58] Billerbeck E, Bottler T, Thimme R (2007). Regulatory T cells in viral hepatitis.. World J Gastroenterol.

[59] Li S, Gowans EJ, Chougnet C, Plebanski M, Dittmer U (2008). Natural regulatory T cells and persistent viral infection.. J Virol.

[60] Mills KH (2004). Regulatory T cells: friend or foe in immunity to infection?. Nat Rev Immunol.

[61] Stoop JN, van der Molen RG, Baan CC, van der Laan LJ, Kuipers EJ (2005). Regulatory T cells contribute to the impaired immune response in patients with chronic hepatitis B virus infection.. Hepatology.

[62] Chou YC, Jeng KS, Chen ML, Liu HH, Liu TL (2005). Evaluation of transcriptional efficiency of hepatitis B virus covalently closed circular DNA by reverse transcription-PCR combined with the restriction enzyme digestion method.. J Virol.

[63] Galibert F, Mandart E, Fitoussi F, Tiollais P, Charnay P (1979). Nucleotide sequence of the hepatitis B virus genome (subtype ayw) cloned in E. coli.. Nature.

[64] Li J, Buckwold VE, Hon MW, Ou JH (1999). Mechanism of suppression of hepatitis B virus precore RNA transcription by a frequent double mutation.. J Virol.

[65] Calvert J, Summers J (1994). Two regions of an avian hepadnavirus RNA pregenome are required in cis for encapsidation.. J Virol.

[66] Biermer M, Puro R, Schneider RJ (2003). Tumor necrosis factor alpha inhibition of hepatitis B virus replication involves disruption of capsid Integrity through activation of NF-kappaB.. J Virol.

[67] Borlak J, Niehof M (2009). HNF4alpha and HNF1alpha dysfunction as a molecular rational for cyclosporine induced posttransplantation diabetes mellitus.. PLoS One.

